# Late ICU admission in liver transplant recipients: ten years experience

**DOI:** 10.1186/2197-425X-3-S1-A898

**Published:** 2015-10-01

**Authors:** F Atar, E Gedik, Ş Kaplan, P Zeyneloglu, A Pirat, M Haberal

**Affiliations:** Baskent University, Ankara, Turkey

## Introduction

Respiratory complications after liver transplantation have been reported to range from 64% to 88%. Liver transplant recipients requiring ICU admission for acute respiratory failure (ARF) is associated with high mortality and morbidity rates [1].

## Objectives

The aim of this study was to evaluate late ICU admission in liver transplant recipients to identify incidences and causes of ARF in the postoperative period and compare those without ARF.

## Methods

We retrospectively screened the data of 173 consecutive adult liver transplant recipients who received their grafts at Başkent University Transplant Center from January 2005 through March 2015. Among them patients admitted to ICU were included for the analysis. ARF was defined as severe dyspnea, respiratory distress, decreased oxygen saturation (< 92%), hypoxemia (PaO2< 60mmHg) or hypercapnia (PaCO2>60mmHg) on room air or requirement of noninvasive or invasive mechanical ventilation. Demographic, clinical and laboratory data were collected. Model for End-stage Liver Disease (MELD), Acute Physiology and Chronic Health Evaluation II (APACHE II) and Sequential Organ Failure Assessment (SOFA) scores at ICU admission and lengths of ICU, hospital stay and mortality were assessed.

## Results

Among the 173 adult liver transplant recipients, 37 (21%) were admitted to ICU, including 22 (60%) with ARF. Mean MELD, APACHE II and SOFA scores of patients with ARF at ICU admission were 22 ± 10, 28 ± 7 and 12 ± 5, respectively. Mean patient age was 47 ± 13 years with 84% males and median time from transplantation to ICU admission was 122 days (0 to 1694). The leading cause of ARF was pneumonia (n = 19, 86%) followed by pulmonary thromboembolism (n = 2, 10%) and cardiogenic pulmonary edema (n = 1, 5%). Patients with ARF had significantly lower levels of albumin (2.9 ± 0 g/dL vs 3.4 ± 1 g/dL) when compared to those without ARF (p = 0.03). Severe sepsis (n = 20, 91% vs n = 5, 33%, p = 0.001) and septic shock (n = 18, 82% vs n = 4, 27%, p = 0.002) were more frequently observed in ARF patients than patients without ARF. Compared to patients without ARF, tracheotomy was more frequently performed in patients with ARF (59% vs 20%, p = 0.018). The length of ICU stay was significantly longer (22.9 ± 22.8 days vs 5.8 ± 3.2 days) and ICU mortality was significantly higher (59% vs 7%) in patients with ARF (p = 0.002, p = 0.001, respectively).

## Conclusions

Acute respiratory failure was detected in 60% of liver transplant recipients with late ICU admission. The leading cause of ARF was pneumonia and patients with ARF had higher requirements of invasive mechanical ventilation and tracheotomy. The length of ICU stay was longer and mortality was higher in patients with ARF compared to those without ARF.
